# Genome-wide identification and analysis of epithelial-mesenchymal transition-related RNA-binding proteins and alternative splicing in a human breast cancer cell line

**DOI:** 10.1038/s41598-024-62681-0

**Published:** 2024-05-23

**Authors:** Yin Mi, Meilian Dong, Xiaoxiao Zuo, Qinchen Cao, Xiaobin Gu, Hailong Mi, Fankai Xiao

**Affiliations:** 1 Department of Radiation Oncology, The First Affiliated Hospital of Zhengzhou University, 1 Jianshe Road, Zhengzhou, 450052 China; 2 Department of Breast Surgery, The First Affiliated Hospital of Zhengzhou University, Zhengzhou, China; 3 Department of Oncology, The First Affiliated Hospital of Zhengzhou University, Zhengzhou, China

**Keywords:** Alternative splicing, Breast cancer, Cell adhesion, Epithelial-mesenchymal transition, Immune escape, Breast cancer, Cancer genetics, Cancer genomics, Metastasis, Tumour biomarkers, Tumour immunology, Cancer genetics, Genome-wide analysis of gene expression, Genome-wide association studies, Bioinformatics

## Abstract

Exploring the mechanism of breast cancer metastasis and searching for new drug therapeutic targets are still the focuses of current research. RNA-binding proteins (RBPs) may affect breast cancer metastasis by regulating alternative splicing (AS) during epithelial-mesenchymal transition (EMT). We hypothesised that during EMT development in breast cancer cells, the expression level of RBPs and the gene AS pattern in the cell were significantly changed on a genome-wide scale. Using GEO database, this study identified differentially expressed RBPs and differential AS events at different stages of EMT in breast cancer cells. By establishing the correlation network of differential RBPs and differential AS events, we found that RBM47, PCBP3, FRG1, SRP72, RBMS3 and other RBPs may regulate the AS of *ITGA6*, *ADGRE5*, *TNC*, *COL6A3* and other cell adhesion genes. By further analysing above EMT-related RBPs and AS in breast cancer tissues in TCGA, it was found that the expression levels of ADAT2, C2orf15, SRP72, PAICS, RBMS3, APOBEC3G, NOA1, ACO1 and the AS of *TNC* and *COL6A3* were significantly correlated with the prognosis of breast cancer patients. The expression levels of all 8 RBPs were significantly different in breast cancer tissues without metastasis compared with normal breast tissues. Conclusively, eight RBPs such as RBMS3 and AS of *TNC* and *COL6A3* could be used as predictors of breast cancer prognosis. These findings need to be further explored as possible targets for breast cancer treatment.

## Introduction

Breast cancer is a common malignant tumour; it has high recurrence, metastasis and mortality rates, and its incidence rate is also increasing yearly. The latest global cancer report shows that breast cancer ranks first among malignant tumours in terms of in number of new cases and second in terms of number of deaths^1^. With the promotion of early breast cancer screening technology and the improvement of comprehensive treatments such as surgery, radiotherapy, chemotherapy and targeted therapy, great progress has been made in the treatment of breast cancer, and the prognosis of patients has significantly improved^2^. However, for metastatic breast cancer, the survival rate is still very low. Due to the lack of clear therapeutic targets, new treatments have been limited over the past few decades, and chemotherapy remains the primary treatment^3^. Therefore, it is still necessary to continue to explore the molecular mechanism of breast cancer metastasis and find drug targets for clinical treatment.

RNA-binding proteins (RBPs) are proteins that regulate gene expression and function by binding RNA. In the process of RNA transcription and posttranscriptional alternative splicing (AS), modification, transport, translation and degradation metabolism, a variety of specific RBPs bind to RNA for transcription and posttranscriptional regulation^4^. Abnormal expression or loss of function of RBPs may lead to the development of various diseases, such as cancer, and affect the metastasis of cancer, making RBPs a potential therapeutic target^5^. Some RBPs, such as lin28, RBM47, RNPC1, and HuR, have been found to have the ability to regulate breast cancer metastasis^6–9^. AS after gene transcription is the main source of protein diversity, which is related to the occurrence and development of many diseases, and RBPs can affect the metastasis of cancer by regulating AS^10–13^. RBPs hnRNPM and RALY have been shown to promote breast cancer metastasis by regulating AS during epithelial-mesenchymal transition (EMT)^14,15^.

We hypothesised that during EMT development in breast cancer cells, the expression level of RBPs and the gene AS pattern in the cell were significantly changed on a genome-wide scale. The abnormal expression of RBPs might affect EMT by regulating AS, thereby affecting the metastasis of breast cancer cells. However, no studies have systematically revealed abnormalities in RBP expression and AS at different stages of EMT in breast cancer.

Therefore, on this basis, this study identified RBPs that were abnormally expressed at different stages of EMT in breast cancer cells and explored their splicing regulatory targets and potential functions in breast cancer metastasis to find new potential therapeutic targets.

## Materials and methods

### Retrieval and processing of public data

We searched the Gene Expression Omnibus (GEO) database with breast cancer and metastasis as keywords. According to the experimental design and the amount of data, we finally selected the GSE172609 dataset (https://www.ncbi.nlm.nih.gov/geo/query/acc.cgi?acc=GSE172609). Six single cell clones were isolated from SUM149PT, a heterogeneous ER-/PR- inflammatory breast cancer line, ranging from epithelial-like (E) to mesenchymal (M1 and M2), including three distinct intermediate states (EM1, EM2, and EM3) in previous studies^16^. Gene expression of EMT markers such as VIM (vimentin), CDH1 (E-cadherin) and ZEB1 was one of the factors that determined the EMT state of each clone. The results for EMT markers across the populations from GSE172609 had been shown in previous studies^16,17^. We also generated the expression amounts of these three genes, with trends similar to those in the original dataset (Supplementary Fig. S1). The above RNA-seq data in different stages of EMT (E, EM1, EM2, EM3, M1, M2) contained in GSE172609 were selected in this study. Three duplicate samples were selected for each stage, and sequencing data of 18 samples were finally obtained for subsequent analysis. Public sequence data files were downloaded from the Sequence Read Archive (SRA). SRA Run files were converted to fastq format with NCBI SRA Tool fastq-dump. The raw reads were trimmed of low-quality bases using a FASTX-Toolkit (v.0.0.13; http://hannonlab.cshl.edu/fastx_toolkit/). Then, the clean reads were evaluated using FastQC (http://www.bioinformatics.babraham.ac.uk/projects/fastqc).

The Cancer Genome Atlas (TCGA) Breast Invasive Carcinoma project data, including gene expression profile and clinical information, were downloaded from the UCSC Xena database (https://xenabrowser.net/datapages/). Splice junction data in browse extensive data format from TCGA were downloaded from Genomic Data Commons Data Portal for alternative splicing identification^18^.

### Read alignment and differentially expressed gene (DEG) analysis

Clean reads were aligned to the GRCh38 genome by HISAT2 (v.2.2.1)^19^. Uniquely mapped reads were ultimately used to calculate read number and reads per kilobase of exon per million fragments mapped (RPKM) for each gene. The expression levels of genes were evaluated using RPKM values. DESeq2 software (v.1.30.1) was used to perform gene differential expression analysis^20^. DESeq2 was used to analyse the differential expression between two or more samples, and the analysis results were used to determine whether a gene was differentially expressed based on the fold change (FC ≥ 2 or ≤ 0.5) and false discovery rate (FDR ≤ 0.05).

### Identification of differentially expressed RBPs (DERBPs)

DERBPs were filtered out from all DEGs according to a catalogue of 2,141 RBPs retrieved from previous reports^4,21–23^.

### WGCNA and module-trait associations

To fully clarify the gene expression pattern, we applied weighted gene coexpression network analysis (WGCNA)^24^ to analyse and cluster the DERBPs. The characteristic genes of each cluster module were used as the representative expression pattern of genes in each module. WGCNA module-trait associations were calculated by a linear mixed effect (LME) model with EMT used as covariates.

### Functional enrichment analysis

Gene Ontology (GO) terms and KEGG pathways were identified using KOBAS 2.0^25^. Hypergeometric tests and Benjamini–Hochberg FDR correction tests were used to define the enrichment level of each term.

### AS analysis

Regulatory alternative splicing (RAS) events were defined and quantified using the Splice sites Usage Variation Analysis (SUVA) pipeline^26^. The different splicing of each group was analysed. The read proportion of the SUVA AS event (pSAR) of each AS event was calculated. The screening criteria for significantly different AS events were P value ≤ 0.05.

### Coexpression analysis

Coexpression analysis was performed for all DERBPs and RAS (pSAR ≥ 50%). Moreover, the Pearson correlation coefficient between DERBPs and RAS was calculated, and DERBP-RAS pairs satisfying the absolute value of the correlation coefficient ≥ 0.9 and P value ≤ 0.01 were screened.

### Establishment of a risk assessment model

To establish a risk assessment model based on coexpressed RBPs and EMT-related RAS/DEGs, univariate Cox regression analysis was used to identify the RBPs and RAS related to prognosis (P ≤ 0.05). Then, least absolute shrinkage and selection operator (LASSO)-penalised Cox regression analysis was performed to further screen prognostic RBPs and RAS using the R packages “survival (v3.5.5)” and “survminer (v0.4.9)”. Cox regression analysis was then used to calculate the coefficient value of RBPs and RAS^27,28^. The risk score was calculated as follows: risk score = Σ(Ci × EXPi), where EXP is the gene expression level and C is the coefficient for the corresponding gene in the Cox model. The autocalculated cut-off of the risk score by the “surv_cutpoint” function from the “survival” R package was used to classify the patients in the training and validation cohorts into high- and low-risk groups. The survival differences between the high- and low-risk groups were compared using the “survival” R package.

### Ethics approval and consent to participate

Since the data used in this study came from the public database, this study was exempt from ethical review. We obtained the public data in accordance with the databases’ ethical guidelines. Data processing was carried out in accordance with the TCGA Human Subject Protection and Data Access Policy.

## Results

### Identification of DERBPs during EMT in breast cancer cells

By differential expression analysis of breast cancer cells at different stages of EMT, we identified a large number of DEGs among the comparison groups (Supplementary Data S1). We found that the number of DEGs decreased first and then increased during the transformation process, and there were fewer DEGs among the comparison groups in the intermediate state (Supplementary Fig. S2A).

**Figure 1:**
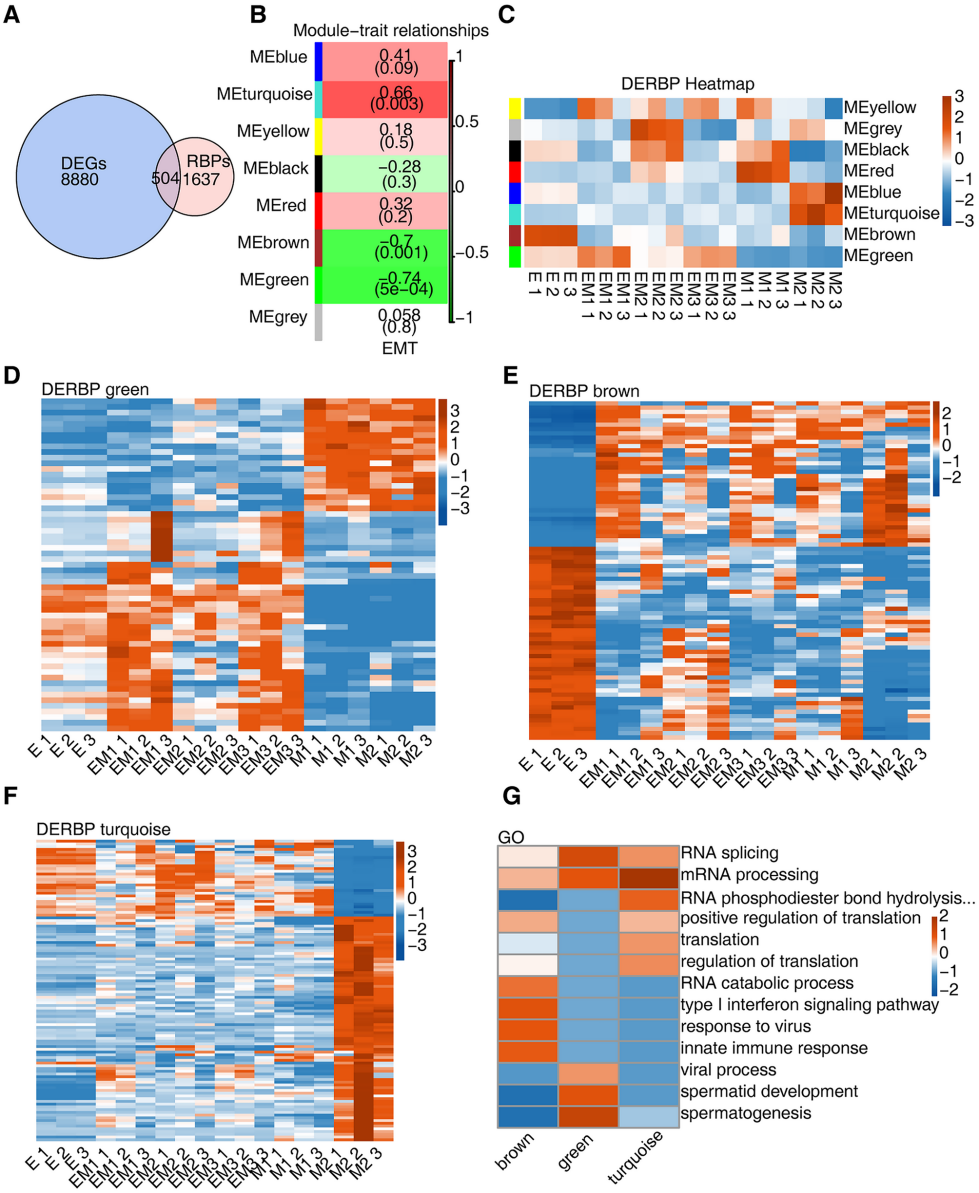
Identification of EMT-related RBPs in a breast cancer cell line. (**A**) Venn diagram showing the overlap of DEGs and RBP genes. (**B**) The correlation between DERBPs in different modules and EMT state. Module-trait associations were computed by a LME model with all factors on the x axis used as covariates. All Pearson’ s correlation value and P values are displayed. (**C**) Heatmap of module eigengenes sorted by average linkage hierarchical clustering. FPKM values were log2-transformed and then median-centred by each gene (color figure online). (**D**–**F**) Heatmap showing the expression profile of DERBPs of green, brown and turquoise module. FPKM values were log2-transformed and then median-centred by each gene (color figure online). (**G**) The top 5 most enriched GO terms were illustrated for DERBP genes in the three modules.The colour scale showing the row-scaled significance (−log10 corrected P value) of the terms.

We combined all identified DEGs and intersected them with known human RBP genes and found that 504 RBP genes were differentially expressed during EMT in breast cancer cells (Fig. 1A)^4^. WGCNA was used to analyse the coexpression relationships among DERBPs (Supplementary Data S2). We found that according to the expression of RBPs in different EMT stages, these genes could be divided into different modules, and the expression of genes in each module was relatively similar at different stages (Supplementary Fig. S2B). Using the WGCNA process, we calculated the correlation between the genes of each module and the EMT state and found that the MEgreen, MEbrown and MEturquoise modules were significantly correlated with the EMT state (Fig. 1B). Among them, the MEgreen module gene was highly expressed in the intermediate state, the MEbrown module gene was highly expressed in the E state cells, and the MEturquoise module gene was highly expressed in the M state cells (Fig. 1C).

RBPs in the MEgreen, MEbrown and MEturquoise modules were further extracted, and a heatmap of expression was drawn. Most of the RBPs in the MEgreen module were highly expressed in the intermediate state (Fig. 1D). Some of the RBPs in the MEbrown module gene were highly expressed in E state cells, while other RBPs were expressed at extremely low levels in this state (Fig. 1E). Most RBPs of MEturquoise module genes were highly expressed in M3 state cells, while a small portion of RBPs were expressed at extremely low levels in M3 state cells (Fig. 1F). These results suggested that the expression level of RBPs might affect the conversion process of EMT in breast cancer.

The genes of MEgreen, MEbrown and MEturquoise were extracted for GO pathway analysis. The results showed that pathways enriched in MEbrown genes mainly included the innate immune response, immune system processes, mRNA processing (Supplementary Fig. S2C). The pathways enriched in MEgreen genes mainly included spermatogenesis, cell differentiation, RNA splicing, mRNA processing. (Supplementary Fig. S2D). The pathways of enriched in MEturquoise genes included mRNA processing and RNA splicing (Supplementary Fig. S2E). We further extracted the common GO functional pathways enriched in MEgreen, MEbrown and MEturquoise genes. The results showed that the MEgreen gene had the highest degree of enrichment in RNA splicing and spermatogenesis pathways, the MEturquoise gene had the highest degree of enrichment in mRNA processing, and the MEbrown gene had the highest degree of enrichment in innate immune pathways (Fig. 1G).

According to the above results, high expression of RBPs in breast cancer cells in the E state might regulate the expression of immune-related genes in cancer cells to achieve immune escape. Breast cancer cells in intermediate state overexpressed RBPs related to splicing regulation and promoted EM progression. Breast cancer cells with M status highly expressed RBPs related to mRNA processing and realised the transformation of the M phenotype.

### Identification of AS during EMT in breast cancer cells

Based on the transcriptome data of 18 breast cancer cell samples at different EMT stages, AS events were analysed according to the use of splicing sites using the SUVA pipeline. Five types of AS events, such as alternative 5' splice site, were identified (Supplementary Fig. S3A).

**Figure 2:**
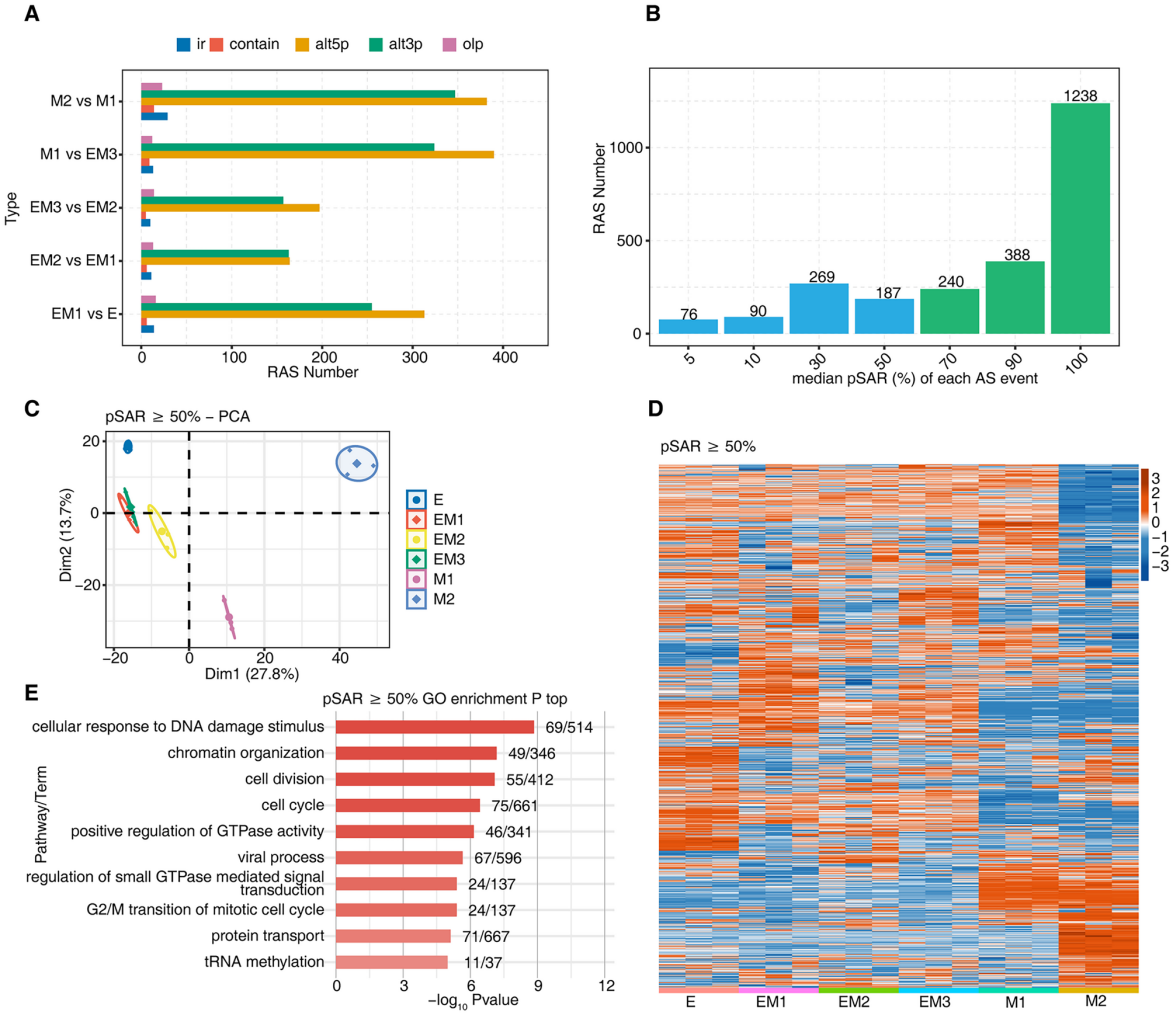
Identification of EMT-related AS in a breast cancer cell line. (**A**) Bar plot showing number of RAS detected by SUVA in each group. (**B**) Bar plot showing RAS with different pSAR. RAS with pSAR ≥ 50% were labeled. (**C**) Principal component analysis based on RAS with pSAR ≥ 50%. The ellipse for each group was the confidence ellipse. (**D**) Heatmap showing the splicing ratio of RAS (pSAR ≥ 50%). Splicing ratio were log2-transformed and then median-centred by each gene (color figure online). (**E**) Bar plot exhibited the most enriched GO biological process results of the RAS with pSAR ≥ 50%.

The SUVA pipeline was used to compare the pSAR used in the same splicing event between the two groups of samples. We identified a large number of AS events, such as those involving alternative 5' splice sites and alternative 3' splice sites, between different comparison groups (Fig. 2A). In addition, by matching the splicing events detected by SUVA to classical splicing events, 10 kinds of splicing events, including a large number of events involving alternative 3’ splice sites, were found (Supplementary Fig. S3B). According to the pSAR used by each differential splicing event, the median pSAR value of the differential AS event was calculated. We found that most of the differential AS events had pSAR values greater than 50% (Fig. 2B). Principal component analysis was performed based on pSAR values of differential splicing events with pSAR ≥ 50% in each sample. The results showed that breast cancer cells at the E, EM1, EM2, EM3, M1 and M2 stages were clustered together. This suggested that these differential AS events can be used to distinguish breast cancer cells at different EMT stages (Fig. 2C).

A heatmap was drawn with pSAR values of differential splicing events with pSAR ≥ 50%. The pSAR values of some splicing events in breast cancer cells at the E, EM and M2 stages were higher than those at other stages (Fig. 2D).

To identify the potential functions of these differential AS events, we extracted the genes responsible for these differential AS events and performed GO and KEGG analyses. GO analysis showed that these genes were enriched in pathways including cellular response to DNA damage stimulus, cell division, cell cycle, positive regulation of GTPase activity, protein transport, tRNA methylation (Fig. 2E). KEGG analysis showed that these genes were enriched in pathways including adherens junction, Epstein-Barr virus infection, fatty acid biosynthesis, ferroptosis, yersinia infection (Supplementary Fig. S3C).

### DERBPs potentially regulated AS associated with cell adhesion in a breast cancer cell line

**Figure 3:**
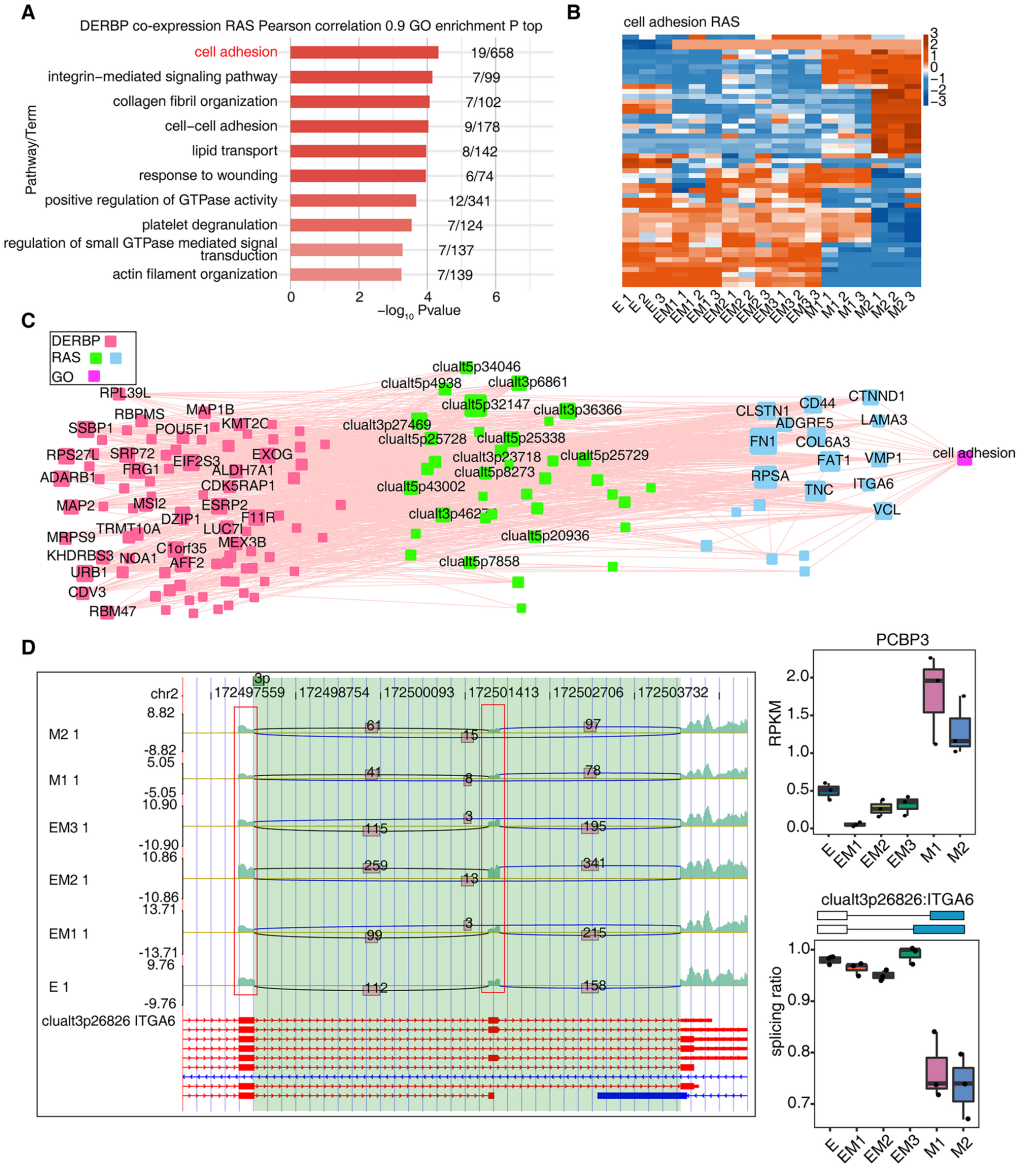
DERBPs potentially regulated AS associated with cell adhesion in a breast cancer cell line (**A**) The most enriched GO biological process results of the coexpressed RAS (pSAR ≥ 50%) potentially regulated by DERBPs. Cutoffs of P value ≤ 0.01 and Pearson coefficient ≥ 0.9 or ≤ − 0.9 were applied to identify the coexpression pairs. (**B**) Heatmap showing the splicing ratio of RAS in cell adhesion pathway. Splicing ratio were log2-transformed and then median-centred by each gene (color figure online). (**C**) Regulatory networks for differential AS events and coexpressed RBPs on genes in the cell adhesion pathway. (**D**) The reads distribution and splicing ratio of clualt3p26826 ITGA6. The expression levels of PCBP3 in breast cancer cells at different EMT stages were showed in the right part.

Given that RBPs can regulate the AS of some genes during EMT in breast cancer, we extracted differential splicing events with pSAR ≥ 50% and RBPs in MEgreen, MEbrown, and MEturquoise modules associated with EMT. By using the expression levels of these RBPs and the pSAR of differential AS events to establish a coexpression relationship, we obtained the AS events potentially regulated by RBPs related to EMT. The genes involved in these differential splicing events were extracted for GO function analysis. We found that these genes were significantly mainly enriched in cell adhesion, the integrin-mediated signalling pathway, lipid transport, positive regulation of GTPase activity (Fig. 3A).

In view of the important role of cell adhesion in EMT and cancer metastasis^29^, we further extracted differential AS events corresponding to genes enriched in cell adhesion pathways. According to their pSAR values, a heatmap was drawn. The pSAR of some differential splicing events was higher in the E and EM stages, while the pSAR of other differential splicing events was higher in the M stage (Fig. 3B).

We constructed regulatory networks for differential AS events and coexpressed RBPs on genes in the cell adhesion pathway and found that 88 RBPs may regulate 37 differential splicing events on 19 cell adhesion pathway genes (Fig. 3C). RBM47, PCBP3, FRG1, SRP72 and other RBPs might regulate AS of *ITGA6*, *ADGRE5*, *TNC* and other genes and affect the EMT process of breast cancer cells (Fig. 3D and Supplementary Fig. S4).

### EMT-related RBPs and AS were significantly correlated with the prognosis of breast cancer patients

**Figure 4:**
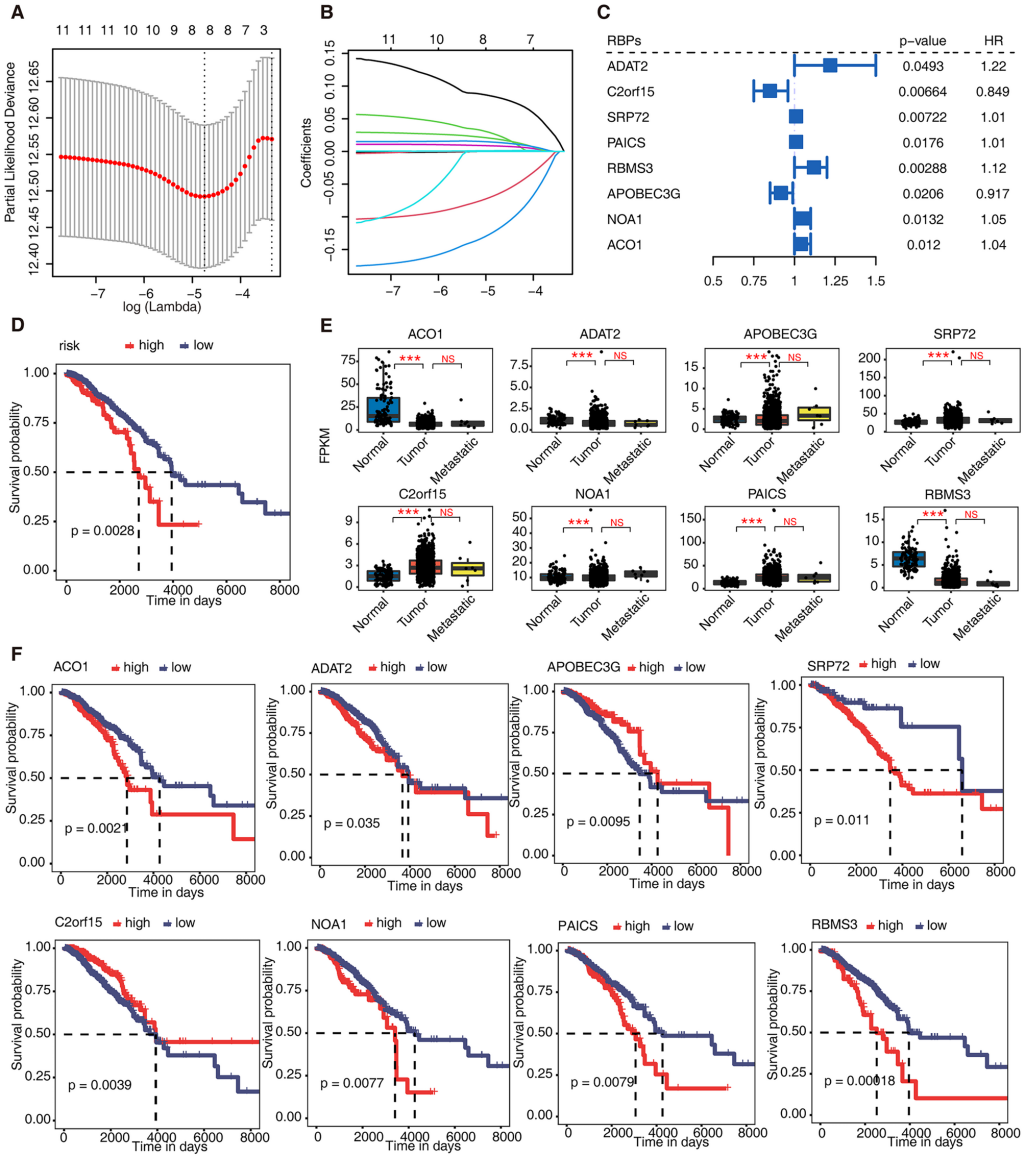
EMT-related RBPs were significantly correlated with the prognosis of breast cancer patients. (**A**) The result of LASSO regression analysis. (**B**) LASSO coefficient profiles of the candidate RBPs by tenfold cross-validation. (**C**) Prognostic value of the candidate RBPs in breast cancer. The HR and P values were calculated using the univariate Cox regression analysis. (**D**) Comparison of overall survival according to the risk score calculated from candidate RBPs. (**E**) The boxplot showing the FPKM of candidate RBPs in Tumour, Metastatic and Normal samples. * ≤ 0.05;** ≤ 0.01;*** ≤ 0.001. (**F**) Relationship between expression level of candidate RBPs and prognosis of breast cancer.

We further downloaded the sequencing data of breast cancer patients and related clinical information from the TCGA database and extracted the expression levels of the above 88 RBPs with regulatory effects. In the RBPs-related analysis, 1216 breast cancer patients were screened (Supplementary Table S1). The median follow-up was 905 days (interquartile range 462–1694 days), with 200 deaths. We constructed a risk model based on the expression levels of these RBPs and found that ADAT2, C2orf15, SRP72, PAICS, RBMS3, APOBEC3G, NOA1, and ACO1 could be used for risk assessment in terms of breast cancer prognosis (Fig. 4A–C). Patients predicted to be at high risk using this model had a significantly worse prognosis (Fig. 4D). We found significant differences in the expression levels of all 8 RBPs in breast cancer tissues without metastasis compared with normal breast tissues. Perhaps due to the small number of metastatic samples, the expression levels of the 8 RBPs in breast cancer tissues with vs. without metastasis were not significantly different (Fig. 4E). Further analysis showed that the expression levels of 8 RBPs in breast cancer tissues were significantly correlated with the prognosis of patients (Fig. 4F).

**Figure 5:**
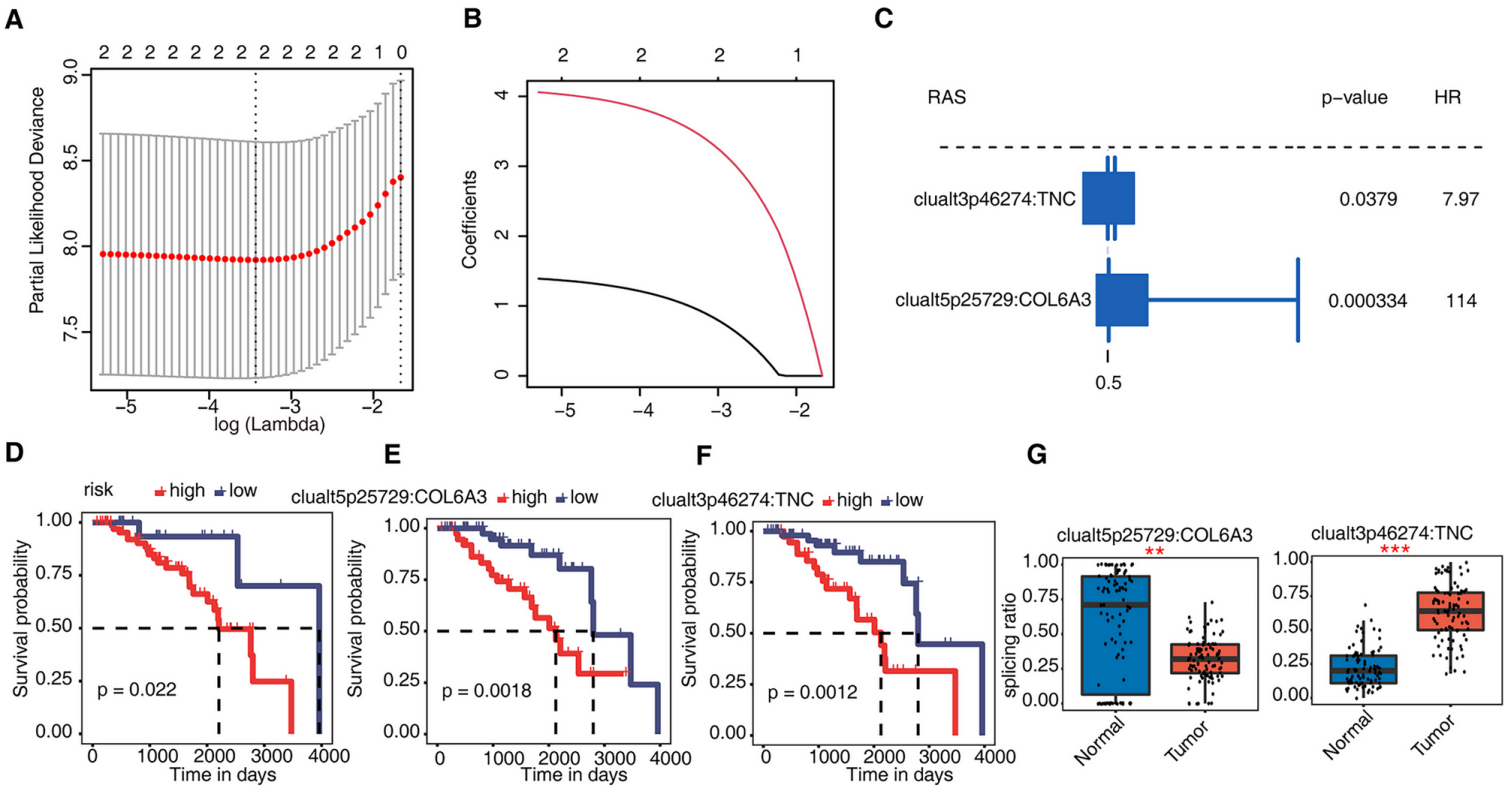
EMT-related AS were significantly correlated with the prognosis of breast cancer patients. (**A**) The result of LASSO regression analysis. (**B**) LASSO coefficient profiles of the candidate AS by tenfold cross-validation. (**C**) Prognostic value of the candidate AS in the breast cancer. The HR and P values were calculated using the univariate Cox regression analysis. (**D**) Comparison of overall survival according to the risk score calculated from candidate AS. (**E**,**F**) Relationship between the pSAR of candidate AS and prognosis of breast cancer. (**G**) The boxplot showing the splicing ratio of clualt5p25729 COL6A3 and clualt3p46274 TNC in Tumour and Normal samples. * ≤ 0.05;** ≤ 0.01;*** ≤ 0.001.

In the AS-related analysis, 90 breast cancer patients were screened (Supplementary Table S2). The median follow-up was 1268 days (interquartile range 774–2129 days), with 26 deaths. We used SUVA to identify differential AS events between breast cancer tissue and normal tissue in TCGA and obtained pSAR values of 37 differential splicing events related to 19 cell adhesion pathway genes. Risk analysis based on pSAR values of splicing events showed that splicing events occurring on *TNC* and *COL6A3* could be used to evaluate breast cancer prognosis (Fig. 5A–C). The analysis found that patients with high-risk differential splicing events had a poor prognosis (Fig. 5D). We found that there were significant differences in the pSAR values of these two splicing events in breast cancer tissue without metastasis compared with normal breast tissue (Fig. 5G). Further analysis showed that the pSAR values of these two differential splicing events in breast cancer tissues were significantly correlated with the prognosis of patients (Fig. 5E–F).

## Discussion

By analysing gene expression and AS in breast cancer cells at different stages of EMT, we identified DERBPs and differential AS events associated with EMT. By establishing the correlation network of differential RBPs and differential AS events, we found that RBM47, PCBP3, FRG1, SRP72, RBMS3 and other RBPs may regulate the AS of *ITGA6*, *ADGRE5*, *TNC*, *COL6A3* and other cell adhesion genes, which may affect the EMT process of breast cancer cells. By analysing DERBPs and differential AS in breast cancer tissues in TCGA, it was found that the expression levels of RBPs such as RBMS3 and the pSAR values of AS occurring on *TNC* and *COL6A3* were significantly correlated with the prognosis of patients.

Abnormal activation of EMT is one of the key mechanisms of tumour metastasis. Abnormal activation of EMT enables primary epithelial cancer cells to acquire mesenchymal properties such as aggressiveness and drug resistance, allowing cancer cells to survive in the circulatory system and eventually colonise distant organs^10^. A variety of morphogenic and environmental signals, such as transforming growth factor-β (TGF-β), WNT, epidermal growth factor and platelet-derived growth factor, inflammatory cytokines, and integrin receptor ligands, are involved in the development of EMT^30^. Recent studies have shown that some RBPs can promote breast cancer metastasis by regulating AS during EMT^14,15^. In the process of EMT in breast cancer cells, the expression level of RBPs and the gene AS pattern on a genome-wide scale may change significantly. Abnormal expression of RBPs may affect the EMT process by regulating AS and then affect the metastasis of breast cancer cells. In this study, we identified these differences in the EMT process to identify RBPs and AS events associated with EMT.

This study revealed that RBPs that are highly expressed in breast cancer cells in the E state may regulate the expression of immune-related genes in cancer cells and achieve immune escape. Van den Eynde et al.^31^ believed that EMT not only enabled tumour cells to acquire the ability to invade and metastasise but also conferred an immunosuppressive phenotype. A previous study revealed that EMT was associated with resistance to immunotherapy in melanoma^32^. Parajuli et al.^33^ found that the RBP Arid5a was significantly upregulated in mesenchymal subtypes of tumours, and the absence of Arid5a in tumour cell lines enhanced antitumour immunity in immune-healthy mice. Arid5a is involved in immune evasion by promoting tumour invasion through gMDSCs and Tregs and inhibiting the recruitment and activation of antitumour effector T cells. Therefore, we believe that some RBPs may be involved in the development of the immunosuppressive phenotype mediated by EMT.

It was found in our study that RBM47, PCBP3, FRG1, SRP72 and other RBPs may regulate the AS of *ITGA6*, *ADGRE5*, *TNC* and other genes and affect the EMT process of breast cancer cells. A previous study^34^ revealed that RBM47 was an EMT-related splicing regulator whose cis-regulatory elements were involved in TJP1 AS. During TGF-β-induced EMT, the expression of the TJP1-α isoform in tumour tissues was significantly increased. The TJP1-α- isoform promotes cell migration by enhancing the assembly of actin stress fibres. ITGA6 is an extracellular integrin receptor that has been found to promote EMT and tumour invasion and metastasis^35,36^. The function of PCBP3, FRG1, SRP72 and other genes in splicing regulation has not been reported, and these findings provide a direction for future research.

We found that the expression of RBMS3 was significantly reduced in both breast cancer tissue and metastatic breast tissue compared to normal tissue. Consistent with our findings, Yang et al.^37^ found that the expression of RBMS3 at the mRNA and protein levels was significantly decreased in breast cancer tissues and cells vs. normal controls. Overexpression of RBMS3 significantly inhibited the proliferation, migration and invasion of breast cancer cells. RBMS3 also significantly inhibited the expression of β-catenin, cyclin D1 and c-Myc proteins in breast cancer cells^37^. In addition, Zhu et al.^38^ found that RBMS3 inhibited the expression of Twist1, which further downregulated matrix metalloproteinase 2 and ultimately played a role in inhibiting breast cancer metastasis.

Similar to our findings, Arafat et al.^39^ reported that AS of the *COL6A3* gene was associated with malignancy in pancreatic ductal adenocarcinoma. Inclusion of exons 3, 4, and 6 was associated with increased mRNA and protein levels of *COL6A3*. The presence of *COL6A3* isoforms and high levels of these isoforms appeared to be associated with tumour stage. Several studies are currently exploring the value of *COL6A3* isoforms in cancer diagnosis and prognosis prediction.

AS a preliminary study, we found some RPBs and AS events that may be associated with EMT. Although this study did not find a direct regulatory relationship between a specific RBP and AS of a specific gene, it provides clues for future research. In the future, some cytological experiments can be designed to verify the effect of a certain RBP on cell migration and metastasis in breast cancer cells, and qPCR can be used to identify the AS of some genes to verify the findings of this study.

## Conclusion

Differentially expressed EMT-related RBPs and differential EMT-related AS events were identified in this study. The expression levels of RBPs such as RBMS3 were significantly correlated with the prognosis of patients, while the AS of *TNC* and *COL6A3* was significantly correlated with the prognosis of patients. These indicators could be used as predictors of breast cancer prognosis. The results of this study provide clues to further verify and reveal the function and mechanism of RBPs and AS in the development of breast cancer metastasis and provide references for the discovery of new biomarkers and therapeutic targets for breast cancer.

### Supplementary Information

Supplementary Figure S1.

Supplementary Figure S2.

Supplementary Figure S3.

Supplementary Figure S4.

Supplementary Data S1.

Supplementary Data S2.

Supplementary Table S1.

Supplementary Table S2.

## Data Availability

The sequencing data used in this study are available at GEO database (https:// www.ncbi.nlm.nih.gov/geo/) under accession code GSE172609. The TCGA Breast Invasive Carcinoma project data were downloaded from the UCSC Xena database (https://xenabrowser.net/datapages/). Splice junction data in browse extensive data format from TCGA were downloaded from Genomic Data Commons Data Portal^16^. All the other data that support the findings of this study are available from the corresponding author upon reasonable request.
