# Supplementary figures and images for: Genome-wide identification and analysis of epithelial-mesenchymal transition-related RNA-binding proteins and alternative splicing in a human breast cancer cell line
Source: Sci Rep. 2024 May 23;14:11753. doi: 10.1038/s41598-024-62681-0 (PMC11116388; doi:10.1038/s41598-024-62681-0)

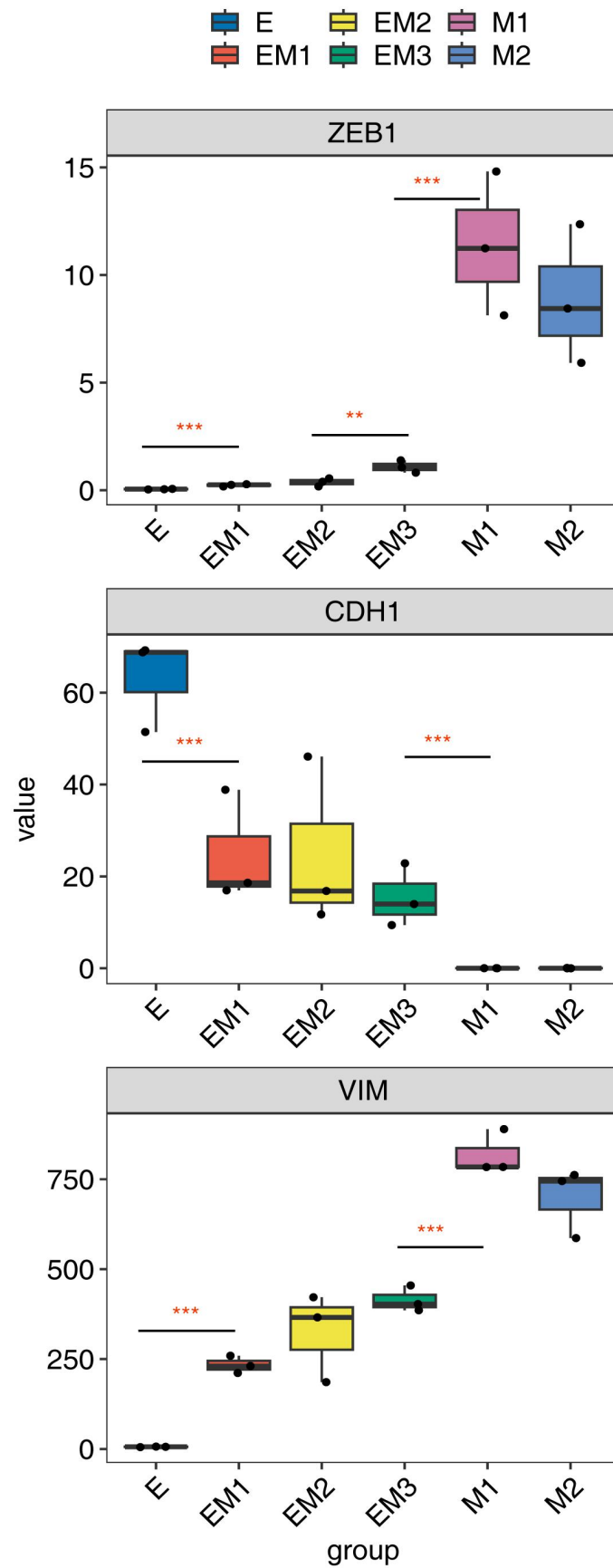

Figure S1. Expression amounts of EMT markers in a breast cancer cell line

Supplement: Supplementary file 1 — Supplementary Figure S1. [file 41598_2024_62681_MOESM1_ESM.pdf]

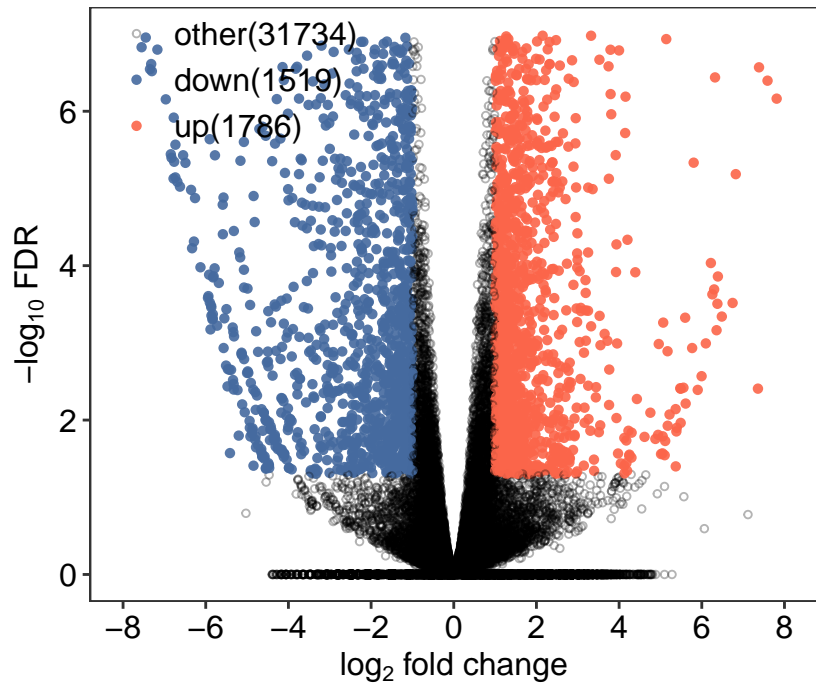

Supplement: Supplementary file 5 — Supplementary Data S1. [file 41598_2024_62681_MOESM5_ESM.zip › EM1_vs_E_DESeq/EM1_vs_E_DEG.pdf]

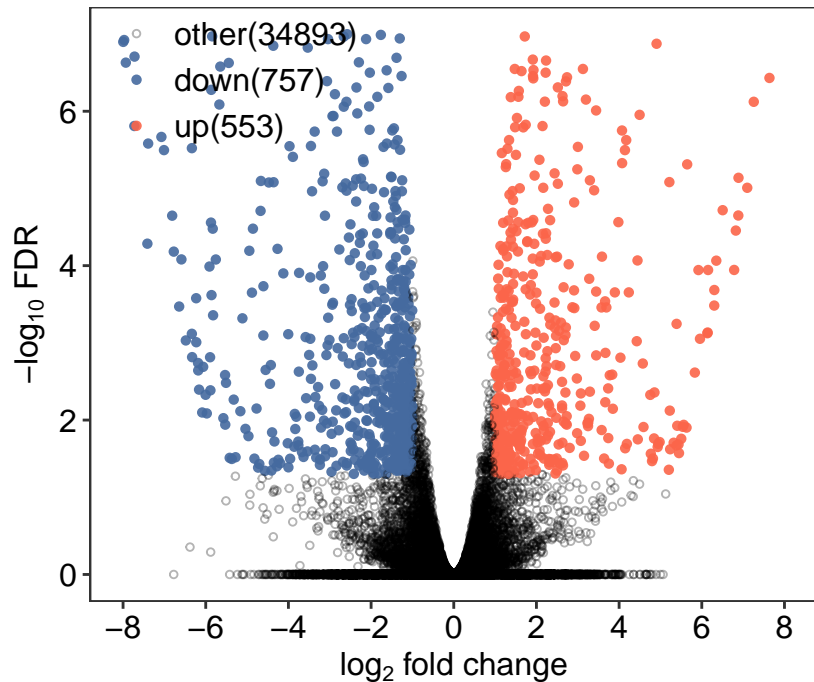

Supplement: Supplementary file 5 — Supplementary Data S1. [file 41598_2024_62681_MOESM5_ESM.zip › EM2_vs_EM1_DESeq/EM2_vs_EM1_DEG.pdf]

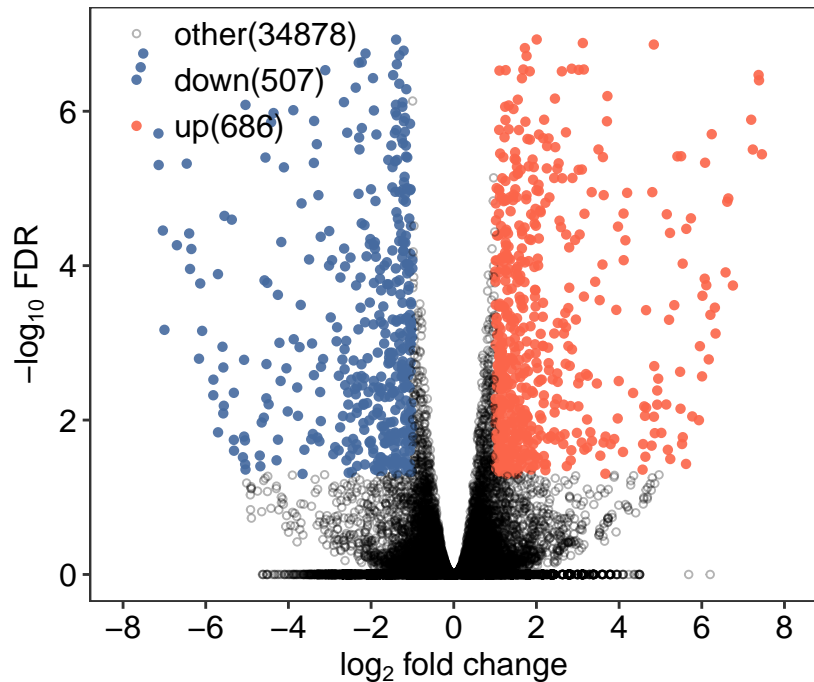

Supplement: Supplementary file 5 — Supplementary Data S1. [file 41598_2024_62681_MOESM5_ESM.zip › EM3_vs_EM2_DESeq/EM3_vs_EM2_DEG.pdf]

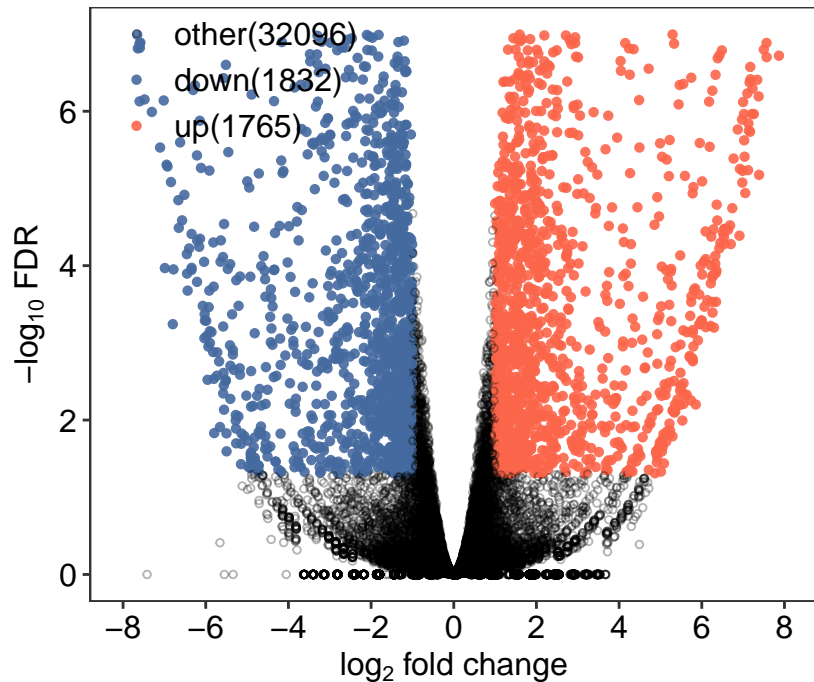

Supplement: Supplementary file 5 — Supplementary Data S1. [file 41598_2024_62681_MOESM5_ESM.zip › M1_vs_EM3_DESeq/M1_vs_EM3_DEG.pdf]

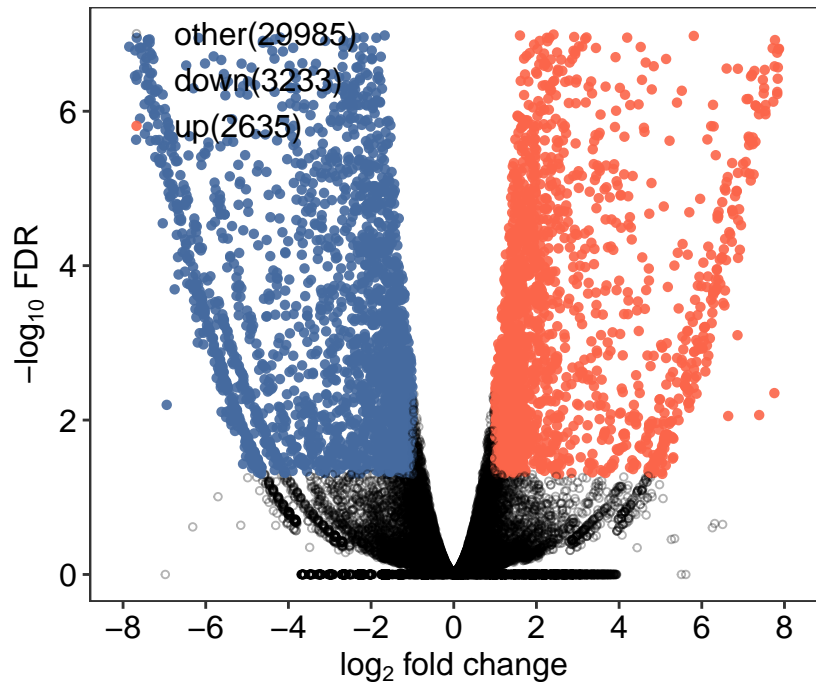

Supplement: Supplementary file 5 — Supplementary Data S1. [file 41598_2024_62681_MOESM5_ESM.zip › M2_vs_M1_DESeq/M2_vs_M1_DEG.pdf]
