# Supplementary material for: Genome-wide identification and analysis of epithelial-mesenchymal transition-related RNA-binding proteins and alternative splicing in a human breast cancer cell line
Source: Sci Rep. 2024 May 23;14:11753. doi: 10.1038/s41598-024-62681-0 (PMC11116388; doi:10.1038/s41598-024-62681-0)
Supplement: Supplementary file 2 — Supplementary Figure S2. [file 41598_2024_62681_MOESM2_ESM.pdf]

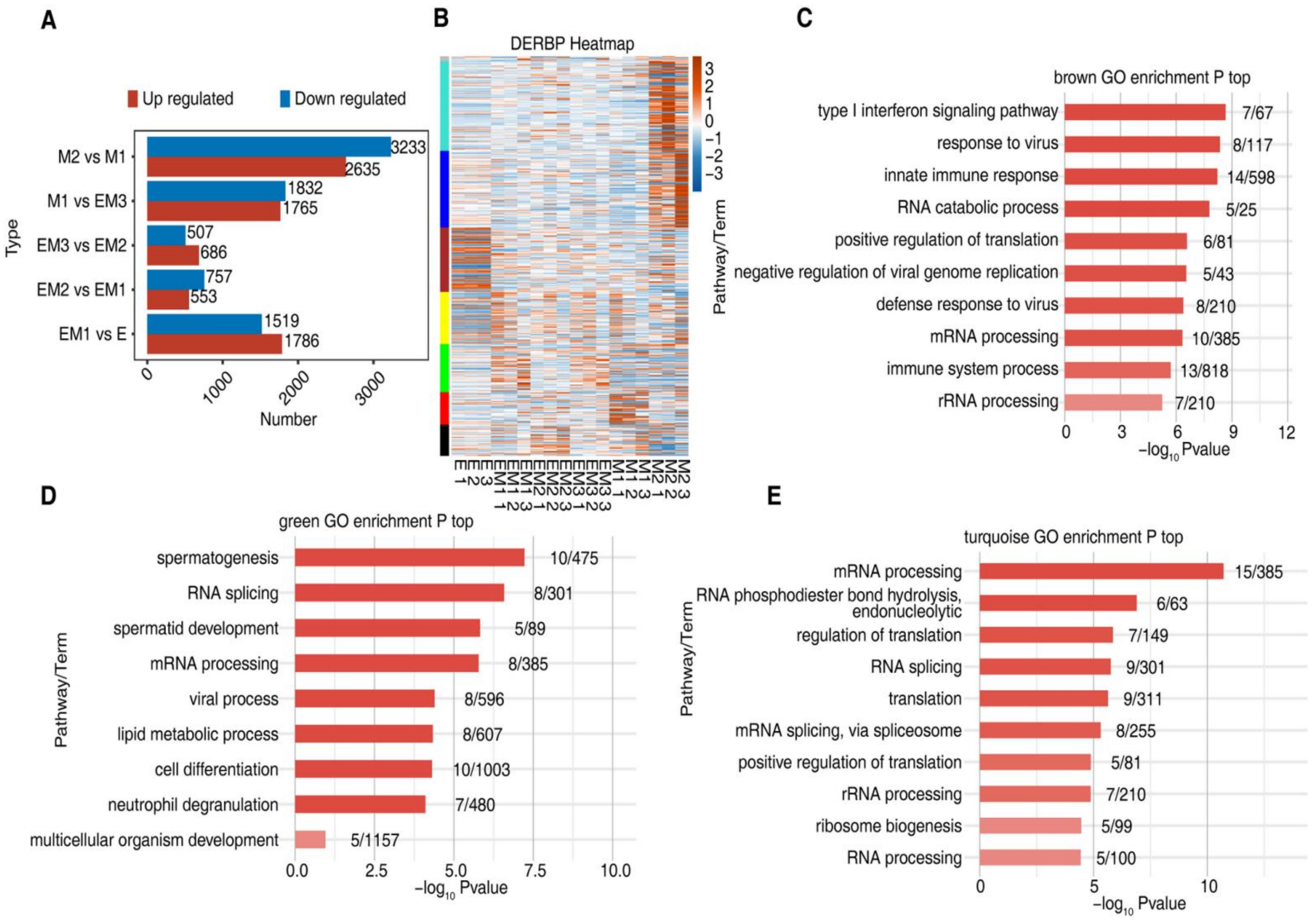

Figure S2. Identification of EMT-related RBPs in a breast cancer cell line

(A) Bar plot showing the number of DEGs among the comparison groups.

(B) Heatmap showing the expression profile of DERBPs by module. FPKM values were log2-transformed and then median-centred by each gene (color figure online).

(C) Bar plot showing the most enriched GO biological process results of brown module.

(D) Bar plot showing the most enriched GO biological process results of green module.

(E) Bar plot showing the most enriched GO biological process results of turquoise module.
