# Supplementary material for: Genome-wide identification and analysis of epithelial-mesenchymal transition-related RNA-binding proteins and alternative splicing in a human breast cancer cell line
Source: Sci Rep. 2024 May 23;14:11753. doi: 10.1038/s41598-024-62681-0 (PMC11116388; doi:10.1038/s41598-024-62681-0)
Supplement: Supplementary file 3 — Supplementary Figure S3. [file 41598_2024_62681_MOESM3_ESM.pdf]

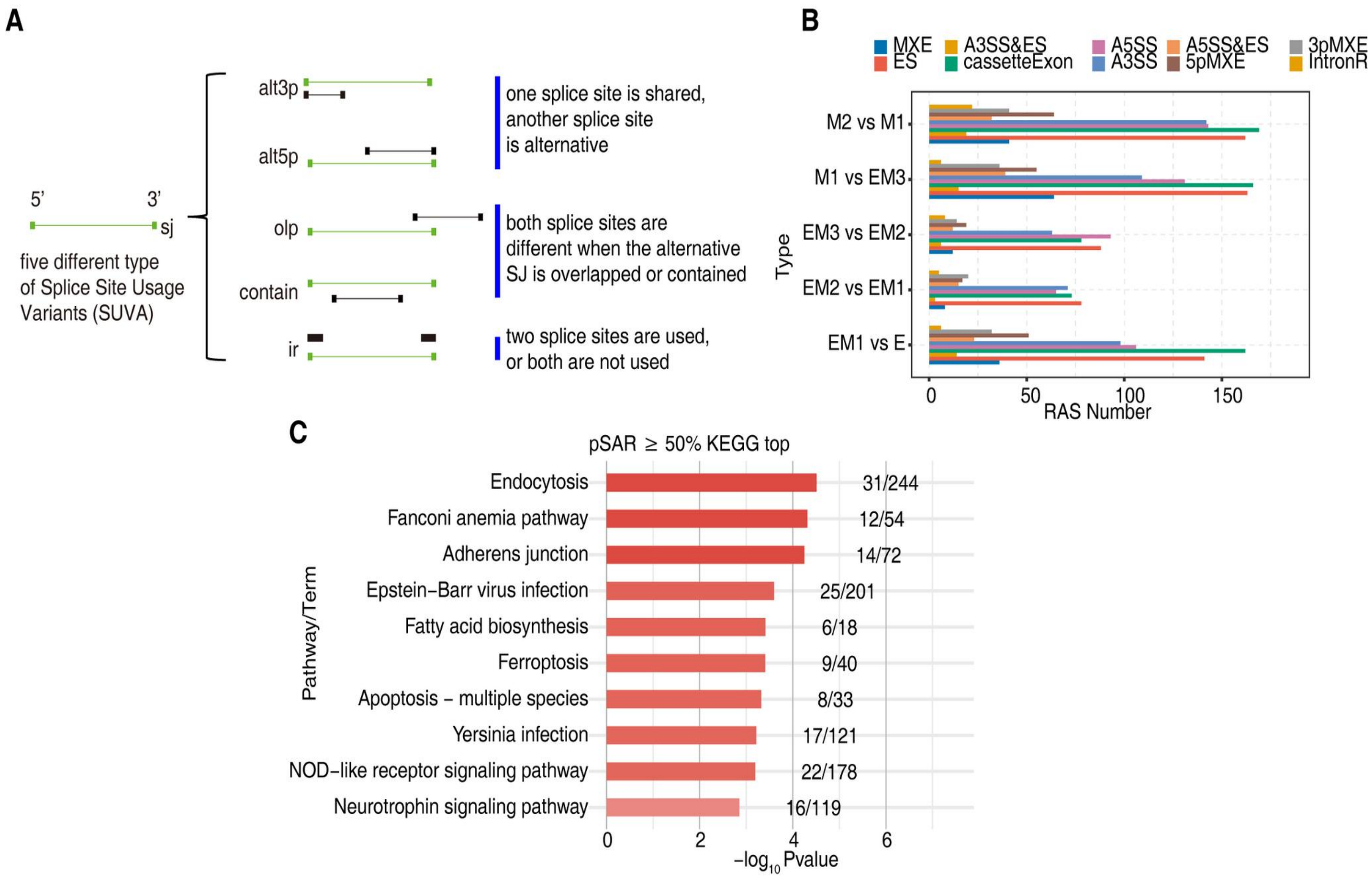

Figure S3. Identification of EMT-related AS in a breast cancer cell line

(A) Schematic diagram of five different types of AS events identified by SUVA.

(B) Quantitative distribution of classical differential splicing events identified at different EMT stages. Splice junction constituting RAS events detected by SUVA were annotated to classical AS event types.

(C) Bar plot showing the top 10 enriched KEGG pathways of the RAS (pSAR ≥ 50%).
