# Supplementary material for: Genome-wide identification and analysis of epithelial-mesenchymal transition-related RNA-binding proteins and alternative splicing in a human breast cancer cell line
Source: Sci Rep. 2024 May 23;14:11753. doi: 10.1038/s41598-024-62681-0 (PMC11116388; doi:10.1038/s41598-024-62681-0)
Supplement: Supplementary file 4 — Supplementary Figure S4. [file 41598_2024_62681_MOESM4_ESM.pdf]

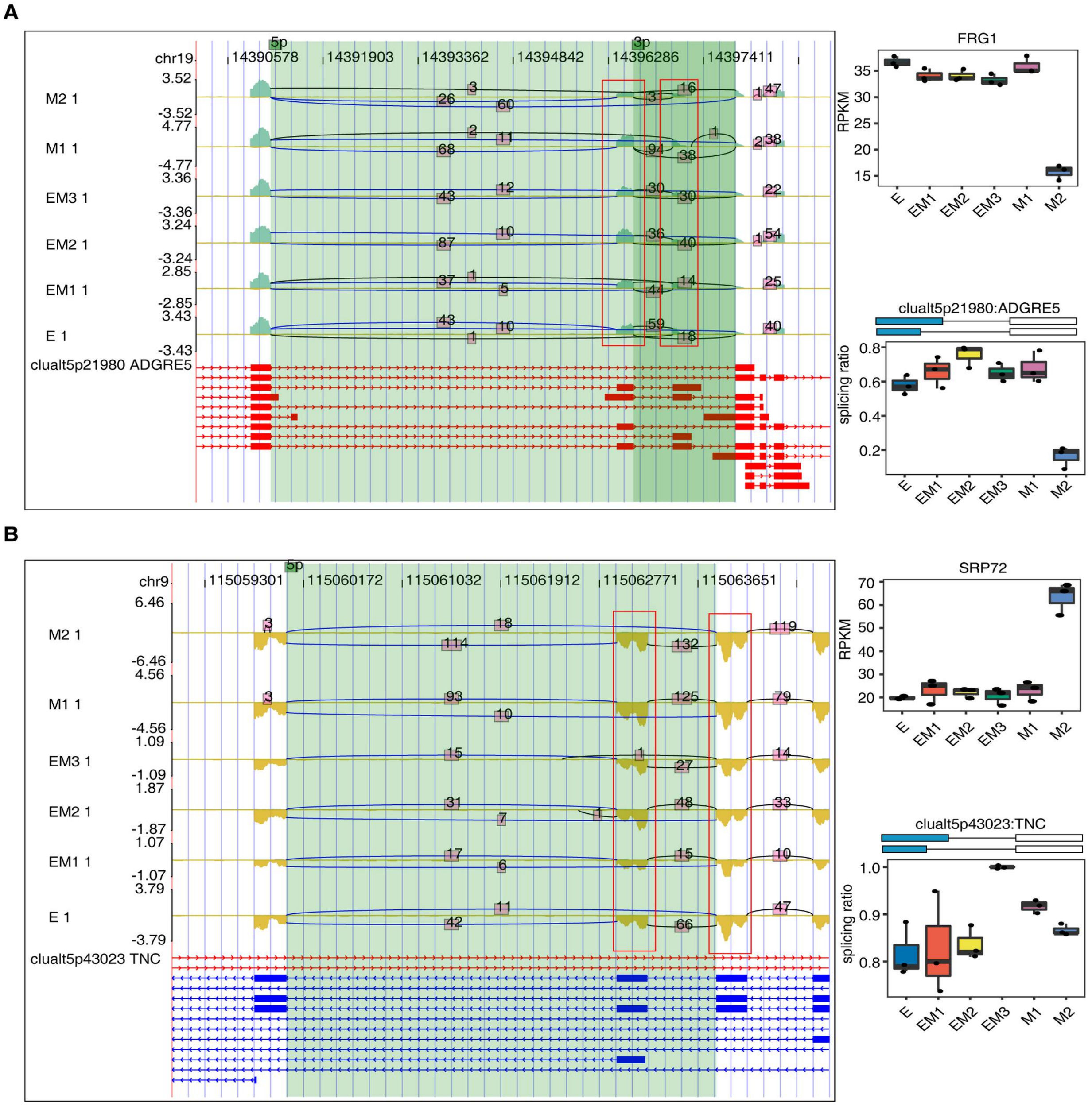

Figure S4. DERBPs potentially regulated AS associated with cell adhesion in a breast cancer cell line

(A) The reads distribution and splicing ratio of clualt5p21980 ADGRE5. The expression levels of FRG1 in breast cancer cells at different EMT stages were showed in the right part.

(B) The reads distribution and splicing ratio of clualt5p43023 TNC. The expression levels of SRP72 in breast cancer cells at different EMT stages were showed in the right part.
