# Supplementary material for: Genome-wide identification and analysis of epithelial-mesenchymal transition-related RNA-binding proteins and alternative splicing in a human breast cancer cell line
Source: Sci Rep. 2024 May 23;14:11753. doi: 10.1038/s41598-024-62681-0 (PMC11116388; doi:10.1038/s41598-024-62681-0)
Supplement: Supplementary file 7 — Supplementary Table S1. [file 41598_2024_62681_MOESM7_ESM.pdf]

Table S1 Characteristics of patients included in the analysis of relationship between RBPs and breast cancer prognosis (n=1216)

Characteristic	N (%)
Age (years)	
mean (SD)	58.3 (13.3)
Gender	
Female	1203 (98.9%)
Male	13 (1.1%)
Race	
White	869 (71.5%)
Black or african american	189 (15.5%)
Asian	62 (5.1%)
Others	1 (0.1%)
Unknown	95 (7.8%)
Histology	
Infiltrating duct carcinoma	874 (71.9%)
Lobular carcinoma	210 (17.3%)
Mixed	69 (5.7%)
Others	63 (5.2%)
Margin status	
Positive	81 (6.7%)
Negative	1042 (85.7%)

Unknown	93 (7.6%)
T stage	
T1	310 (25.5%)
T2	706 (58.1%)
T3	150 (12.3%)
T4	47 (3.9%)
TX	3 (0.2%)
N stage	
N0	563 (46.3%)
N1	416 (34.2%)
N2	132 (10.9%)
N3	82 (6.7%)
NX	23 (1.9%)
M stage	
M0	1021 (84.0%)
M1	24 (2.0%)
MX	171 (14.1%)
Tumour stage	
I	202 (16.6%)
II	693 (57.0%)
III	275 (22.6%)
IV	22(1.8%)

Unknown	24 (2.0%)
Neoadjuvant treatment	
Yes	14 (1.2%)
No	1200 (98.7%)
Unknown	2 (0.2%)
Radiation therapy	
Yes	594 (48.8%)
No	461 (37.9%)
Unknown	161 (13.2%)
