# Supplementary material for: Genome-wide identification and analysis of epithelial-mesenchymal transition-related RNA-binding proteins and alternative splicing in a human breast cancer cell line
Source: Sci Rep. 2024 May 23;14:11753. doi: 10.1038/s41598-024-62681-0 (PMC11116388; doi:10.1038/s41598-024-62681-0)
Supplement: Supplementary file 8 — Supplementary Table S2. [file 41598_2024_62681_MOESM8_ESM.pdf]

Table S2 Characteristics of patients included in the analysis of relationship between differential AS and breast cancer prognosis (n=90)

Characteristic	N (%)
Age (years)	
mean (SD)	55.6 (13.3)
Gender	
Female	89 (98.8%)
Male	1 (1.1%)
Race	
White	82 (91.1%)
Black or african american	6 (6.7%)
Asian	1 (1.1%)
Histology	
Infiltrating duct carcinoma	69 (76.7%)
Lobular carcinoma	6 (6.7%)
Mixed	12 (13.3%)
Others	3 (3.3%)
Margin status	
Positive	3 (3.3%)
Negative	77 (85.6%)
Unknown	10 (11.1%)
T stage	

T1	23 (25.6%)
T2	53 (58.9%)
T3	10 (11.1%)
T4	4 (4.4%)
N stage	
N0	40 (44.4%)
N1	36 (40.0%)
N2	9 (10.0%)
N3	4 (4.4%)
NX	1 (1.1%)
M stage	
M0	84 (93.3%)
M1	1 (1.1%)
MX	5 (5.6%)
Tumour stage	
I	15 (16.7%)
II	54 (60.0%)
III	20 (22.2%)
IV	1 (1.1%)
Neoadjuvant treatment	
Yes	1 (1.1%)
No	89 (98.9%)

Radiation therapy	
Yes	41 (45.6%)
No	27 (30.0%)
Unknown	22 (24.4%)
